# Cholesterol Is a Regulator of CAV1 Localization and Cell Migration in Oral Squamous Cell Carcinoma

**DOI:** 10.3390/ijms24076035

**Published:** 2023-03-23

**Authors:** Nyein Nyein Chan, Manabu Yamazaki, Satoshi Maruyama, Tatsuya Abé, Kenta Haga, Masami Kawaharada, Kenji Izumi, Tadaharu Kobayashi, Jun-ichi Tanuma

**Affiliations:** 1 Division of Oral Pathology, Department of Tissue Regeneration and Reconstruction, Faculty of Dentistry & Graduate School of Medical and Dental Sciences, Niigata University, Niigata 951-8514, Japan; 2 Division of Reconstructive Surgery for Oral and Maxillofacial Region, Faculty of Dentistry & Graduate School of Medical and Dental Sciences, Niigata University, Niigata 951-8514, Japan; 3 Oral Pathology Section, Department of Surgical Pathology, Niigata University Hospital, Niigata 951-8520, Japan; 4 Division of Biomimetics, Department of Oral Health Science, Faculty of Dentistry & Graduate School of Medical and Dental Sciences, Niigata University, Niigata 951-8514, Japan

**Keywords:** caveolin-1, cell polarization, cholesterol, migration, oral squamous cell carcinoma

## Abstract

Cholesterol plays an important role in cancer progression, as it is utilized in membrane biogenesis and cell signaling. Cholesterol-lowering drugs have exhibited tumor-suppressive effects in oral squamous cell carcinoma (OSCC), suggesting that cholesterol is also essential in OSCC pathogenesis. However, the direct effects of cholesterol on OSCC cells remain unclear. Here, we investigated the role of cholesterol in OSCC with respect to caveolin-1 (CAV1), a cholesterol-binding protein involved in intracellular cholesterol transport. Cholesterol levels in OSCC cell lines were depleted using methyl-β-cyclodextrin and increased using the methyl-β-cyclodextrin-cholesterol complex. Functional analysis was performed using timelapse imaging, and CAV1 expression in cholesterol-manipulated cells was investigated using immunofluorescence and immunoblotting assays. CAV1 immunohistochemistry was performed on surgical OSCC samples. We observed that cholesterol addition induced polarized cell morphology, along with CAV1 localization at the trailing edge, and promoted cell migration. Moreover, CAV1 was upregulated in the lipid rafts and formed aggregates in the plasma membrane in cholesterol-added cells. High membranous CAV1 expression in tissue specimens was associated with OSCC recurrence. Therefore, cholesterol promotes the migration of OSCC cells by regulating cell polarity and CAV1 localization to the lipid raft. Furthermore, membranous CAV1 expression is a potential prognostic marker for OSCC patients.

## 1. Introduction

Cholesterol constitutes 30–40% of the lipid component of the plasma membrane [1]. As cancer cells proliferate rapidly, they require high levels of cholesterol for membrane biogenesis and other cellular functions, such as cell signaling [2]. Therefore, cancer cells reprogram cholesterol metabolism by increasing cholesterol influx via lipid receptors [3] or by upregulating de novo biosynthesis of cholesterol via the mevalonate pathway [4], whereas normal cells strictly regulate the cholesterol content of the plasma membrane [5].

Derangement of cholesterol metabolism and its role in tumor progression have been observed in various types of cancers [2]. Cholesterol supplementation in prostate cancer cells enhances cell survival via the PI3K/Akt pathway, but it has a lower effect on normal prostate epithelial cells [6]. Moreover, cholesterol serves as a mitogen for cell proliferation in intestinal stem cells by increasing cholesterol biosynthesis [7]. Cholesterol reprogramming is also observed in oral squamous cell carcinoma (OSCC), with higher cellular cholesterol levels in OSCC tissues than in normal oral mucosa in the same patient [8]. In addition, one of the lipid receptors, CD36, is overexpressed in OSCC and participates in the proliferation and migration of OSCC cells [9]. However, the serum lipid profile, including cholesterol level, was reported to be lower in OSCC patients than in healthy controls [10,11], suggesting a possibility that cancer cells demand and utilize serum cholesterol and lipids for membrane formation and cancer progression [12,13]. Therefore, cellular cholesterol metabolism is vital for carcinogenesis and cancer progression rather than serum cholesterol level [14]. Moreover, cholesterol-lowering agents, such as statins, can inhibit OSCC progression in several ways. Simvastatin decreases the proliferation of oral cancer cells by inhibiting cell-cycle-regulated protein DNA methyltransferase I [15]. Xu et al. reported that pitavastatin can be used as an anticancer drug, as it downregulates MET signaling in oral and esophageal cancers [16]. Thus, cholesterol is suggested to play an important role in the carcinogenesis and progression of OSCC. However, the direct effect of cellular cholesterol levels on OSCC development and pathogenesis has rarely been reported.

Cellular cholesterol metabolism involves various processes, including cholesterol influx, efflux, esterification, and de novo synthesis, in which the metabolism depends on intracellular cholesterol trafficking [17]. In addition to the endolysosomal pathway, caveolin-1 (CAV1), a major structural protein of caveolae in lipid rafts [18], directly binds to cholesterol and transports it between intracellular organelles and caveolae of the plasma membrane [19,20,21] through receptor-independent endocytosis [22]. In OSCC, CAV1 is overexpressed [23,24] and is linked to carcinogenesis, tumor progression, lymph node metastasis, chemoresistance, and poor prognosis [25,26,27,28]. Although intracellular cholesterol and CAV1 levels are increased in OSCC cells, the role of CAV1 with respect to cellular cholesterol levels in the progression of OSCC remains unclear.

Therefore, we hypothesized that cellular cholesterol levels affect the biological activities of OSCC cells by regulating CAV1 expression. In this study, we investigated the functional changes in OSCC cells with respect to cellular cholesterol levels and the underlying mechanisms involving CAV1 expression. In this study, we found that cholesterol manipulations modified CAV1 localization and cell polarity in OSCC cells. Moreover, membranous CAV1 expression could be a potential prognostic biomarker for patients with OSCC.

## 2. Results

### 2.1. Cholesterol Influences the Morphology of OSCC Cells and Promotes Their Migration

**Figure 1:**
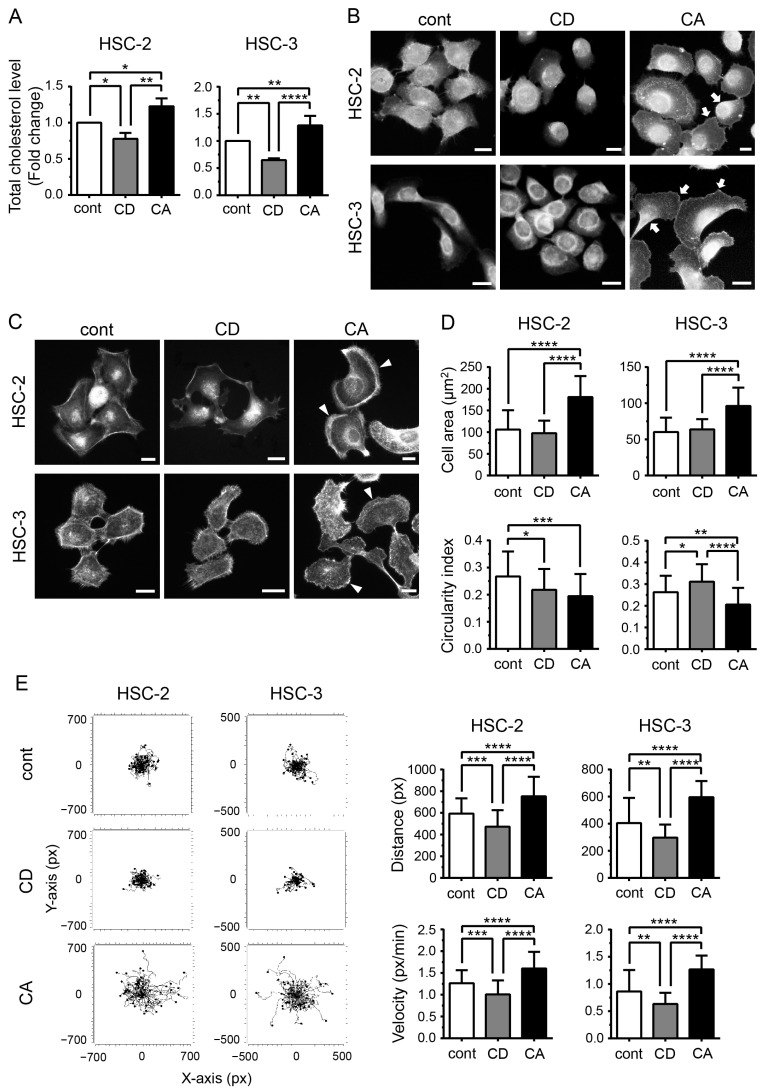
Cholesterol influences cell morphology and promotes cell migration. (**A**) Quantification of total cellular cholesterol levels using Amplex^TM^ Red cholesterol assay. Open boxes indicate control (cont); gray-shaded boxes indicate cholesterol-depleted (CD) cells; solid boxes indicate cholesterol-added (CA) cells. Triplicate results of cholesterol levels represent fold change as mean ± SD (compared to control). * *p* < 0.05, ** *p* < 0.01, and **** *p* < 0.0001. (**B**) Fluorescence staining using a filipin III cholesterol probe. Scale bars, 20 µm. Arrows indicate high filipin III signaling at the periphery of the cells. (**C**) Fluorescence images of F-actin visualized by rhodamine-phalloidin staining to evaluate cellular morphology. Scale bars, 20 µm. Arrowheads indicate the development of a lamellipodia-like structure. (**D**) Bar graphs showing cellular area and circularity index (N ≥ 50) expressed as mean ± SD. * *p* < 0.05, ** *p* < 0.01, *** *p* < 0.001, and **** *p* < 0.0001. (**E**) Cell-tracking plots of timelapse imaging for 8 h (N ≥ 50). Distance and velocity are analyzed using chemotaxis and migration tools (ibidi) and expressed as mean ± SD. ** *p* < 0.01, *** *p* < 0.001, and **** *p* < 0.0001.

To investigate the role of cholesterol in OSCC pathogenesis, we depleted or increased cholesterol levels in cells and observed the viability of cells at different time points. Methyl-β-cyclodextrin (MβCD) is widely used for cholesterol depletion because of its fast extractability, compatibility with live cells, and reversibility [29]. In contrast, the MβCD-cholesterol complex enriches cholesterol in cells [30]. However, the vulnerability of the cells to cholesterol depletion by MβCD depends on its concentration, the incubation duration, and the cell type [30]. In the present study, the viability of treated and untreated cells was comparable up to 8 h, although the HSC-3 cells treated with MβCD for 24 h exhibited a drastic decrease in viability (Figure S1, Supplementary Materials). Thus, we analyzed cellular changes and cell migration after 4 and 8 h of treatment, respectively. Total cholesterol levels were significantly decreased in the MβCD-treated cells (*p* < 0.05 in HSC-2, *p* < 0.01 in HSC-3) and increased in the cells treated with the MβCD–cholesterol complex (*p* < 0.05 in HSC-2, *p* < 0.01 in HSC-3) compared with the control cells (Figure 1A), indicating that cholesterol manipulation was successfully performed. Filipin III signals were detected around the nuclei in the control and cholesterol-depleted (CD) cells, indicating the presence of cholesterol; however, no signals were observed in the plasma membrane (Figure 1B). Meanwhile, in the cholesterol-added (CA) cells, the signals increased in the plasma membrane area in both cell lines (Figure 1B, arrows), suggesting that the cholesterol enrichment procedure used in the present study influenced cholesterol content in the plasma membrane. Moreover, we noticed that increased cellular cholesterol levels changed the shape of the cells. Although most control and CD cells were polygonal in shape, the CA cells were fan-shaped, accompanied by the development of lamellipodia-like structures (Figure 1C, arrowheads). Image analysis revealed that in the case of CA cells, the area was significantly increased (*p* < 0.0001 in both HSC-2 and HSC-3), whereas the circularity index was reduced (Figure 1D), indicating that cholesterol addition promoted cell stretching. Since morphological changes are fundamental processes in cell migration, we tracked the cells using timelapse imaging. Migratory parameters, such as distance and velocity, were significantly increased in CA cells (*p* < 0.0001) and decreased in CD cells (*p* < 0.001 in HSC-2, *p* < 0.01 in HSC-3) in both cell lines (Figure 1E).

### 2.2. Cholesterol Leads to Cell Polarity by Promoting Asymmetric Membranous Localization of CAV1

**Figure 2:**
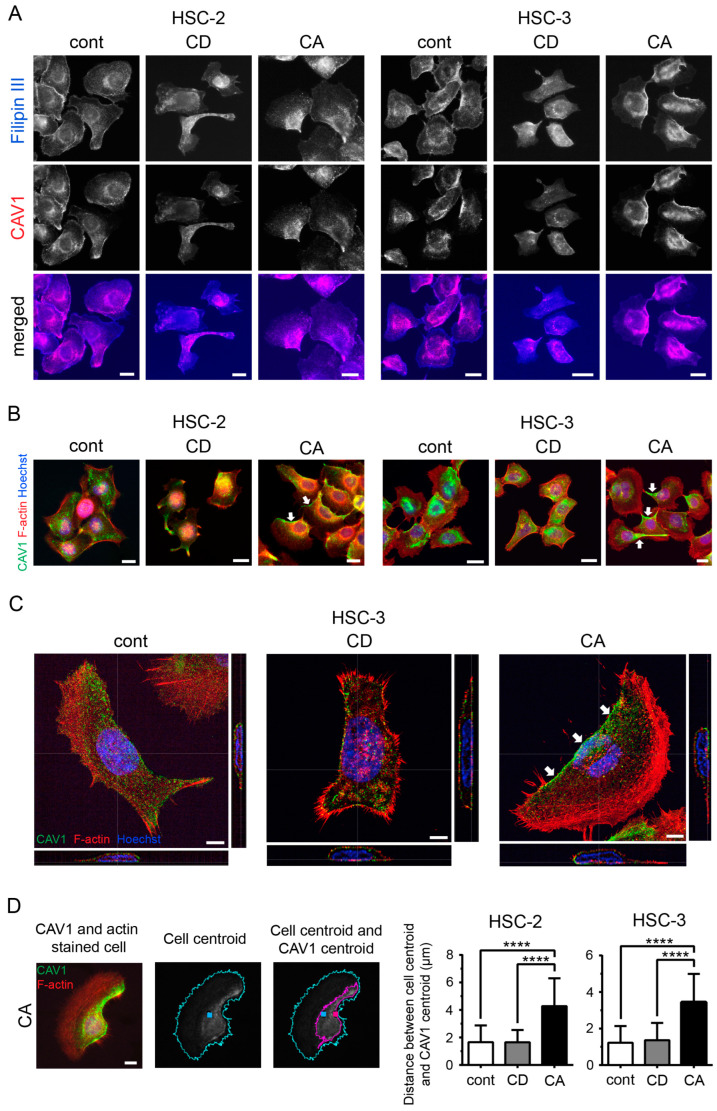
Cholesterol promotes asymmetric membranous localization of CAV1. (**A**) Colocalization of filipin III and CAV1. Blue, filipin III; red, CAV1. Scale bars, 20 µm. (**B**) Fluorescence staining for CAV1 and F-actin. Green, CAV1; red, F-actin; blue, Hoechst. Scale bars, 20 µm. Arrows indicate the asymmetric localization of CAV1. (**C**) CAV1 distribution assessed using confocal microscopy. Green, CAV1; red, F-actin; blue, Hoechst. Scale bars, 5 µm. Arrows indicate the asymmetric localization of CAV1. (**D**) Assessment of CAV1 distribution by centroid position. Cell centroid (blue square) and CAV1 centroid (red square) of CAV1- and actin-stained cells (N ≥ 50) were determined using ImageJ. The distance between the two points was measured using ImageJ software. Scale bar, 10 µm. Bar graphs represent the distance as mean ± SD. **** *p* < 0.0001.

To explore the mechanism of cholesterol-regulated cell migration, we focused on the localization of CAV1, a cholesterol-binding protein [20]. In the combined fluorescence images, filipin III and CAV1 signals were similarly distributed in all samples (Figure 2A), suggesting a close relationship between cholesterol and CAV1. In particular, one-sided distribution of both signals was observed in the CA cells, whereas it was attenuated in the CD cells (Figure 2A). Double staining of CAV1 and F-actin revealed that CAV1 localized to the center of the cell in the control and CD cells, whereas CAV1 was asymmetrically localized close to one side of the cell periphery in CA cells (Figure 2B, arrows). Confocal microscopy revealed that CAV1 was primarily localized in the plasma membrane under all three conditions; CAV1 was uniformly distributed in the plasma membrane in the control and CD cells (Figure 2C). In contrast, in the CA cells, CAV1 was asymmetrically distributed in the plasma membrane on the opposite side of lamellipodia (Figure 2C, arrows), and lamellipodia were devoid of CAV1. The asymmetric distribution of CAV1 in the CA cells was also confirmed by the increased distance between the cell centroid and CAV1 centroid (Figure 2D). These data suggest that cholesterol regulates CAV1 distribution.

**Figure 3:**
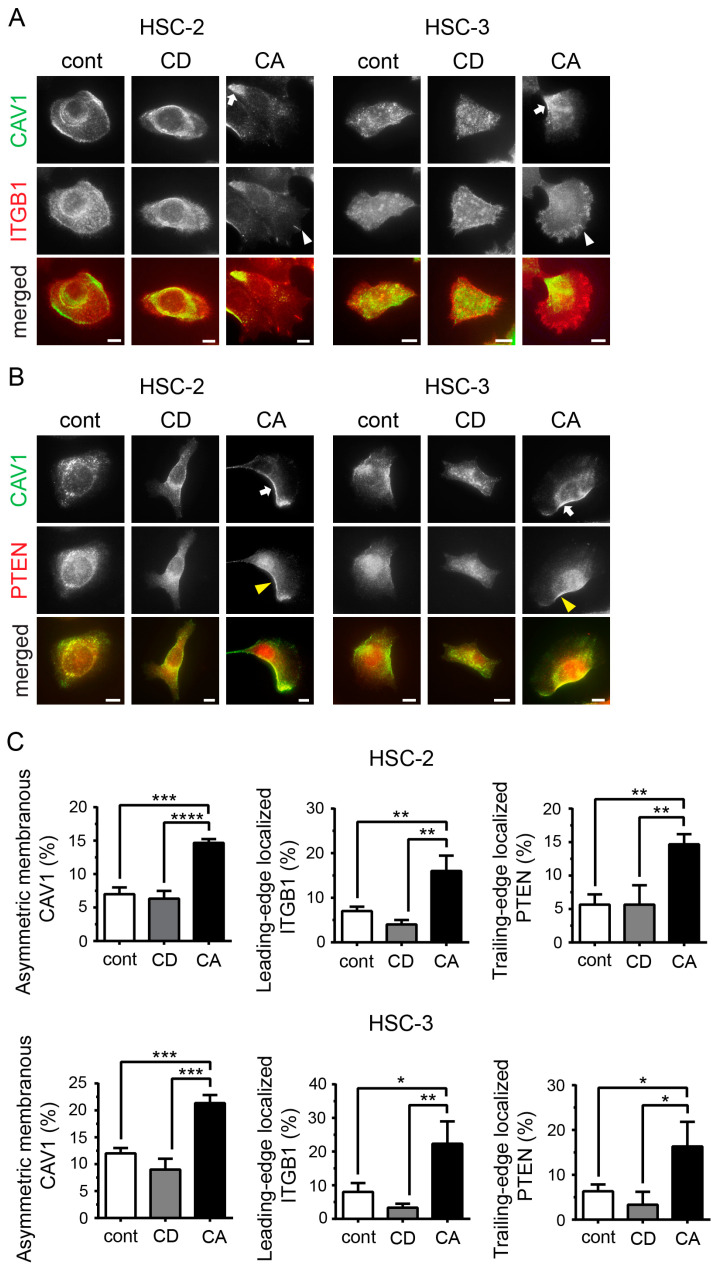
Cholesterol endows cell polarity by asymmetric membranous localization of CAV1. (**A**) Combined immunofluorescence staining of CAV1 and integrin β1 (ITGB1). Green, CAV1; red, ITGB1. Scale bars, 10 µm. White arrows indicate asymmetric membranous localization of CAV1 at the trailing edge of polarized cells; white arrowheads indicate ITGB1 at the cell leading edge in polarized cells. (**B**) Combined immunofluorescence staining of CAV1 and PTEN. Green, CAV1; red, PTEN. Scale bars, 10 µm. White arrow, asymmetric membranous CAV1 at the trailing edge of polarized cells; yellow arrowhead, PTEN at the trailing edge in polarized cells. (**C**) Percentage of polarized cells as per the asymmetric localization of CAV1, ITGB1, and PTEN. Bar graphs show triplicate results of the percentage of polarized cells expressed as mean ± SD. * *p* < 0.05, ** *p* < 0.01, *** *p* < 0.001, and **** *p* < 0.0001.

Since the asymmetric organization of proteins in the plasma membrane is characteristic of cell polarity [31], we investigated CAV1 distribution and cell polarity. As integrins, including ITGB1, translocate to the leading edge [32] and PTEN moves laterally and posteriorly [31] in a migrating cell, ITGB1 and PTEN were used as markers of the leading edge and trailing edge of the polarized cells, respectively. Combined immunofluorescence staining of CAV1 and ITGB1, as well as of CAV1 and PTEN, revealed asymmetric localization of CAV1 on the opposite side of ITGB1 (Figure 3A) and on the same side as PTEN (Figure 3B) in polarized cells. Thus, CAV1 was located at the trailing edge along the plasma membrane in polarized cells. Furthermore, the ratio of polarized cells substantially increased among the CA cells in both cell lines (Figure 3C), indicating that cell migration promoted by cholesterol supplementation was related to front–rear cell polarity.

### 2.3. Cholesterol Increases the CAV1 Level in Cold Triton X-100 (TX)-Resistant Lipid Raft Region

**Figure 4:**
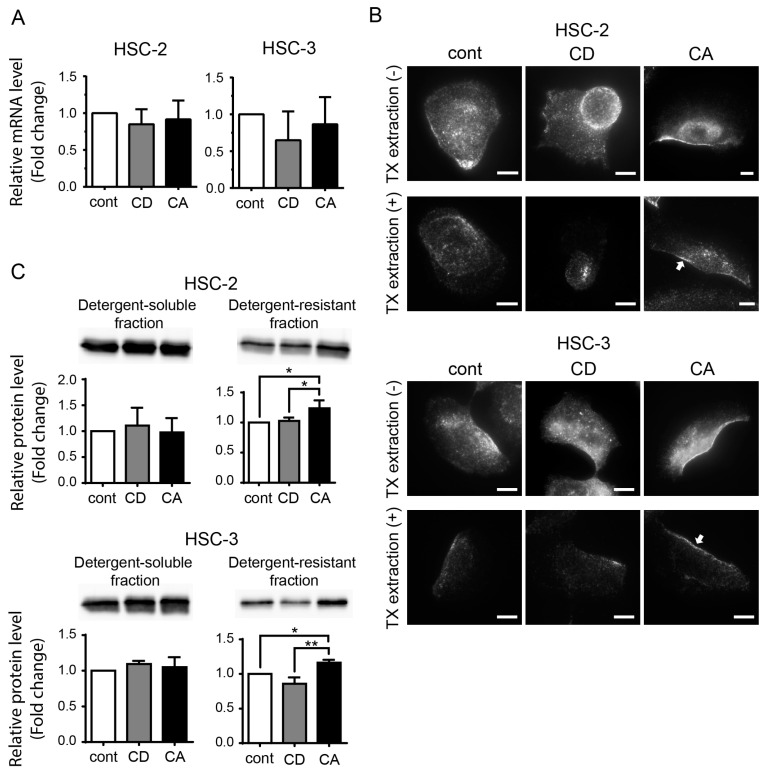
Cholesterol manipulation changes the CAV1 protein level in the cold Triton X-100 (TX)-resistant lipid raft region. (**A**) Quantitative RT-PCR for CAV1. Triplicate results are shown as mean ± SD. (**B**) Immunofluorescence staining for CAV1 in cells with or without TX pre-extraction in HSC-2 (upper) and HSC-3 (lower). Scale bars, 10 µm. Arrow indicates asymmetric membranous localization of CAV1 in TX pre-extracted cells. (**C**) CAV1 protein level in detergent-soluble fraction and detergent-resistant fraction in HSC-2 (upper) and HSC-3 (lower). Coomassie blue stain was used as the loading control. Bar graphs show triplicate results of relative protein level expressed as mean ± SD. * *p* < 0.05, **p < 0.01.

Next, we assessed whether cholesterol manipulation affected CAV1 expression at the mRNA and protein levels. CAV1 mRNA expression was not altered in the control and cholesterol-manipulated samples (Figure 4A). To determine CAV1 protein levels in the lipid raft, we applied the cold TX pre-extraction method [33], by which detergent-soluble (non-lipid raft) proteins were removed and the detergent-resistant (lipid raft) proteins remained in the cells. We observed that CAV1 signals were markedly decreased in the pre-extracted cells (Figure 4B, lower panels). In the pre-extracted samples, the control and CD cells exhibited a dramatic decrease in CAV1 signals, whereas the CA cells retained high-level asymmetric membranous CAV1 signals (Figure 4B, arrows). These results indicate that cholesterol promotes CAV1 anchoring in the lipid rafts of the plasma membrane. Moreover, the CAV1 level in the detergent-resistant fraction was considerably increased in the CA cells compared to that in the control and CD cells (Figure 4C, Figure S2 in Supplementary Materials). However, significant changes were not observed in the detergent-soluble fraction.

### 2.4. Membranous CAV1 Expression in OSCC Tissue Specimens Is Correlated with the Aggressiveness of the Tumor and Poor Clinical Outcomes

**Figure 5:**
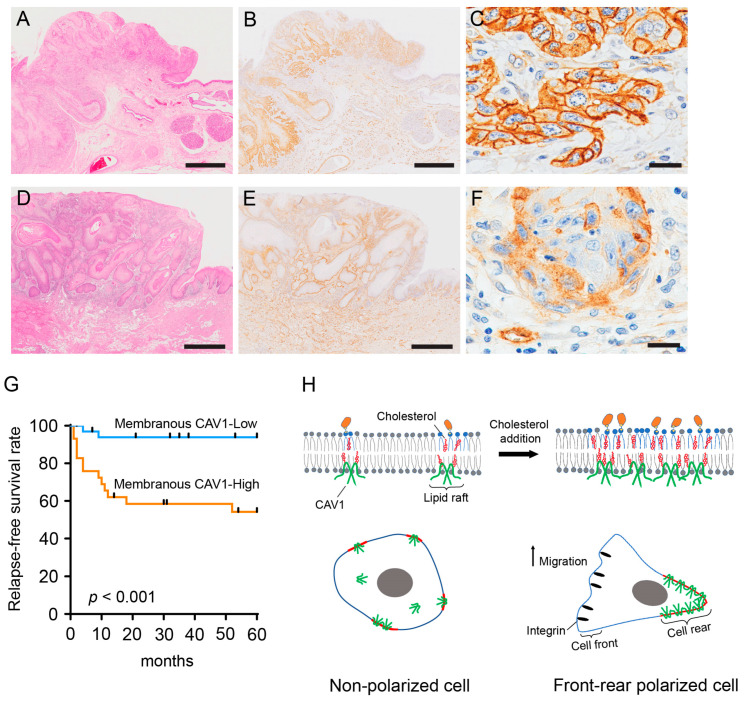
Membranous CAV1 expression in oral squamous cell carcinoma (OSCC) tissue and its correlation with relapse-free survival. (**A**–**C**) Representative photomicrograph of the tumor with high membranous CAV1 expression (21%). (**D**–**F**) Representative photomicrograph of the tumor with low membranous CAV1 expression (6%). (**A**,**D**) H&E staining. (**B**,**C**,**E**,**F**) Immunostaining for CAV1. (**C**) and (**F**) are higher-magnification images of (**B**,**E**), respectively. Scale bars, 1 mm (**A**,**B**,**D**,**E**) and 20 µm (**C**,**F**). (**G**) Kaplan–Meier curve showing relapse-free survival of OSCC patients based on membranous CAV1 expression. High membranous CAV1 expression is significantly associated with poor relapse-free survival (*p* < 0.001). (**H**) Schematic diagram showing the conclusions of this study. Cholesterol addition increases the cholesterol level in the plasma membrane and the CAV1 level in the lipid raft. Meanwhile, the cell promotes cell migration by changing its shape from polygonal to fan-shaped; polarized morphology with trailing edge localization of CAV1.

We examined the clinicopathological relationship and prognostic impact of CAV1 expression levels on the survival of OSCC patients. CAV1 positivity was enhanced in OSCC nests compared with that in non-cancerous oral epithelia (Figure 5A–F). In OSCC cells, CAV1 signals were linearly detected in the plasma membrane and granularly in the cytoplasm (Figure 5C,F). Moreover, the staining intensity in the plasma membrane was higher than that in the cytoplasm. However, the degree of CAV1 membrane positivity was heterogeneous across tumors; in some tumors, CAV1 membrane positivity was observed throughout the tumor (Figure 5B–C), whereas in other tumors, it was observed only in limited areas (Figure 5E–F).

Next, since CAV1 was more intensely distributed in the plasma membrane in the CA cells, as shown in Figure 2B,C, we quantified the area (%) of the OSCC cells with stronger or comparable membranous CAV1 expression compared with that of endothelial cells, and 63 tumors were divided into two groups with a cutoff value of 15% or greater.

Consequently, 29 tumors (46%) were classified into the group with high membranous CAV1 expression, and 34 (54%) were classified into the group with low membranous CAV1 expression. The correlation between membranous CAV1 expression and the clinicopathological parameters is shown in Table 1. High membranous CAV1 expression was significantly associated with age (*p* < 0.01), lymph node metastasis (*p* < 0.01), clinical stage (*p* < 0.01), and regional recurrence (*p* < 0.01). Moreover, OSCC patients with high membrane expression of CAV1 had lower relapse-free survival (*p* < 0.001) (Figure 5G). Next, we performed univariate and multivariate Cox regression analyses of clinicopathological variables, including CAV1 membrane expression, to determine the potential independent prognostic factors for relapse-free survival. The results revealed that the mode of invasion (*p* < 0.01) and membranous CAV1 expression (*p* < 0.01) were significant variables in the univariate and multivariate Cox regression analyses (Table 2).

## 3. Discussion

The present study demonstrates that cellular cholesterol levels affect the migratory ability of OSCC cells by regulating cell polarity. We revealed that cholesterol-induced cell polarization is characterized by the trailing-edge membranous localization of CAV1, which might be initiated by increased CAV1 in the lipid raft. We also showed that membranous CAV1 expression in OSCC cells is a potential prognostic factor. This study highlights the relationship between cellular cholesterol levels and membranous localization of CAV1, indicating that it potentiates OSCC progression and metastasis.

We showed that cellular cholesterol levels were related to changes in cell morphology and migration. In particular, cholesterol addition resulted in morphological changes in cells, with the development of lamellipodia and an increase in cell size in OSCC cells. Furthermore, cholesterol addition and depletion induced an increase and decrease in OSCC cell migration, respectively. The importance of cholesterol in cell shape and cell migration has been widely studied with respect to membrane cholesterol depletion and replenishment [34,35]. For instance, cholesterol depletion by MβCD induces morphological changes, such as loss of lamellipodia and filopodia, which decrease cell migration [34,35], whereas cholesterol replenishment after depletion restores normal morphological characteristics and cell migration capacity in breast cancer cells [35]. However, the effects of cholesterol supplementation on cellular function have rarely been reported. Qin et al. reported that cholesterol-loaded mouse macrophages exhibit larger cell sizes than control macrophages and develop lamellipodia [36], which is similar to our results. They discovered that cellular cholesterol changes cell shape by increasing pinocytic activity and activating Rac1, the major protein for lamellipodia development; however, cholesterol-loaded macrophages remained polygonal [36]. Nagao et al. reported that cholesterol load suppresses the migration ability of macrophages by interfering with Rho activation [37]. A similar result was reported for tendon-derived stem cells, wherein cholesterol addition disturbed the G0/G1 cell cycle and inhibited directional cell migration [38]. On the contrary, in the present study, cholesterol-added OSCC cells showed fan-shaped morphology with a decreased circularity index, which is interpreted as polarized cells [39] and elevated migration ability. Therefore, we assumed that such cell activation by cholesterol addition may be characteristic of malignant tumor cells.

Our results suggest that CAV1 plays an important role in cholesterol-induced cell polarization. We found that cholesterol addition increased the number of polarized cells exhibiting asymmetric CAV1 distribution at the trailing edge of the plasma membrane. CAV1 localization at the trailing edge of polarized cells has been observed in a variety of cells, such as endothelial cells [40,41,42], mouse embryonic fibroblasts [43], mouse neuronal cells [44], and prostate cancer cells [45]. CAV1 promotes cell polarization via its inhibitory effects on other proteins [42], such as Rac and Cdc42, which induce cell protrusion [46]. That is, CAV1 localization on one side of the plasma membrane potentially causes an induction in lamellipodia and focal adhesion molecules including ITGB1 on the other side [42]. Beardsley et al. demonstrated the role of CAV1 in cell polarity and migration using CAV1 RNA interference; the suppression of CAV1 attenuates cell polarization and directional migration in endothelial cells [42]. In addition, cholesterol reportedly has a significant influence on the sequestration of integrin α_v_β_3_ in a model membrane system [47]. Thus, cholesterol could initiate the segregation of CAV1 and integrins on the plasma membrane, resulting in cell polarization.

Moreover, the increase in CAV1 levels in lipid rafts due to cholesterol addition may be related to CAV1 polarization. We demonstrated that cholesterol addition increased CAV1 protein levels in the detergent-resistant (lipid raft) fraction, whereas the detergent-soluble (non-lipid raft) fraction was less affected; lipid-raft-associated CAV1 was localized at the trailing edge in the CA cells. In the plasma membrane, cholesterol preferentially organizes sphingolipids to form a lipid raft, also called the liquid-ordered domain, in which highly ordered lipid hydrocarbon chains are more tightly packed than the surrounding lipid bilayer [48,49]. Because cholesterol serves as a glue in the tightly organized lipid rafts, the melting temperature of the lipid rafts is higher than that of the non-raft membranes [50]. Therefore, lipid-raft-associated proteins are resistant to TX extraction at low temperatures [48]. CAV1 is a lipid-raft-associated protein [48] that participates in the formation of caveolar lipid rafts [51,52]. Genetic ablation of CAV1 completely eradicates caveolae in mouse embryonic fibroblasts and peritoneal macrophages [52]. Caveolar lipid rafts are considered the major signaling machinery for cell polarity, as their rear-side localization assists directional migration in endothelial [40] and neuronal cells [44]. Moreover, increased CAV1 levels in lipid rafts increase the number of caveolae in the plasma membranes of caveolin cDNA-transfected lymphocytes [53]. Thus, the relationship between cholesterol, CAV1, and caveolae formation is important for cell polarization and migration. Therefore, we assumed that cholesterol initiates cell polarization and promotes migration by upregulating the lipid-raft-associated CAV1 at the trailing edge (Figure 5H), although we could not specify CAV1 localization to caveolae. However, the molecular mechanisms underlying the cell polarity conferred by lipid-raft-associated CAV1 remain unclear, and future studies are warranted.

Apart from cholesterol treatment regulated by MβCD and MβCD–cholesterol complex, other cholesterol-manipulating methods also affect the CAV1 protein level and cell migration. Similar to our findings, high levels of low-density lipoprotein stimulate the development of the actin cytoskeleton in human endothelial cells and raise CAV1 protein levels in lipid rafts without altering overall CAV1 protein levels [54]; however, that study did not examine cell migration. Fluvastatin also affects cytoskeletal organization and inhibits cell migration on fibronectin-coated surfaces in rat vascular smooth muscle cells [55], comparable to our findings. Thus, cellular cholesterol levels may affect CAV1 protein levels in lipid rafts and regulate cell motility without being affected by manipulation techniques. However, activation of cells may vary between cell lines. Contrary to our findings, mevastatin triggers the EGF-Rac1 signaling pathway in primary human keratinocytes by lowering the CAV1 protein level, which promotes cell motility [56]. 

Membranous expression of CAV1 is a possible predictive marker for worse clinical outcomes in patients with OSCC. In the present study, OSCC with high membranous CAV1 expression exhibited an advanced clinical stage, increased regional metastasis, and shorter relapse-free survival. CAV1 is overexpressed in both the membrane and cytoplasm of OSCC cells [23,24,26,57]. Overexpression of CAV1 is associated with regional metastasis [23,25] and poor survival [25,26], despite different scoring methods for CAV1 immunohistochemistry; however, previous studies have not focused on membranous CAV1 expression. Importantly, membranous CAV1 expression has been recognized as a marker for brain tumors with high-grade malignancies [58]. In astroglial tumors, cytoplasmic dot-like, cytoplasmic and membrane, and intense membrane CAV1 staining are observed in grade II, III, and IV tumors, respectively [58], suggesting that CAV1 membrane expression is an indicator of the aggressiveness of tumors, such as astrocytomas and OSCC. In addition, based on our results, we speculated that membranous CAV1 expression is a surrogate indicator for cellular cholesterol levels. However, tissue-based quantification of cholesterol was not possible because fresh tissue was unavailable in the present study. Further studies are necessary to confirm this hypothesis.

The present study has several limitations. First, we did not investigate whether other cholesterol manipulation methods, such as the use of statins for cholesterol depletion and low-density lipoprotein for cholesterol addition, that have more physio-pharmacological significance would have the same effects. Second, the direct mechanistic relationship and molecular mechanisms between CAV1 and cholesterol-induced cellular changes have not been fully validated. It is also necessary to clarify whether CAV1 reciprocally regulates cellular cholesterol levels. These issues should be addressed in future research. Third, since this work is a preliminary examination of the impact of cellular cholesterol on the activities of OSCC cells, we only evaluated planar cell movement on coverslips. Therefore, planar and vertical migration of cholesterol-manipulated cells will be confirmed in the future using 3D models because OSCC cell migration and invasion also depend on the tumor microenvironment, which can be explored in 3D models [59,60], and are related to the underlying substrates [61].

In conclusion, we found that elevated cellular cholesterol levels in OSCC cells induce an aggressive phenotype by regulating CAV1 in the lipid rafts. Moreover, membranous CAV1 expression in tissue specimens has been suggested to be a reliable marker for the prognosis of OSCC. Further studies determining the correlation between cellular cholesterol levels and CAV1 expression could help in the development of effective treatment strategies for OSCC.

## 4. Materials and Methods

### 4.1. Cell Lines

HSC-2, a well-differentiated OSCC cell line, and HSC-3, a poorly differentiated OSCC cell line, developed from the metastatic lymph nodes of the floor of mouth and tongue cancer, respectively, were obtained from the Riken BRC Cell Bank (Tsukuba, Japan). The cells were maintained in minimum essential medium (MEM) (Thermo Fisher Scientific, Waltham, MA, USA) supplemented with 10% fetal bovine serum (FBS) (Thermo Fisher Scientific, Waltham, MA, USA) in a humidified atmosphere with 5% CO_2_/95% air at 37 °C.

### 4.2. Tissue Samples

We included 63 patients who visited the Niigata University Hospital for surgical treatment of primary OSCC between 2006 and 2019. The experimental protocol for analyzing the surgical material was reviewed and approved by the Ethical Board of the Niigata University Graduate School of Medical and Dental Sciences (approval number: 2018-0228). All experiments were conducted in accordance with the guidelines of the Declaration of Helsinki. The median age of the patients was 72 years (range: 29–92 years); 36 patients were male, and 27 were female. Clinical stage was determined according to the Union for International Cancer Control TNM classification system, 8th edition [62]. Serial sections were prepared for hematoxylin and eosin (H&E) and immunohistochemical staining as described in Section 4.12.

### 4.3. Reagents and Antibodies

MβCD (C4555) and cholesterol (C3045) were purchased from Sigma-Aldrich (St. Louis, MO, USA). Filipin III, a cholesterol probe, was obtained from Cayman Chemical Co. (Ann Arbor, MI, USA). The primary antibodies used for immunofluorescence assay were as follows: anti-CAV1 (1:400; D46G3 rabbit IgG mAb; Cell Signaling Technology, Danvers, MA, USA), anti-PTEN (1:100; clone 17A, mouse IgM mAb, Lab Vision, South San Francisco, CA, USA), and anti-integrin β1 (ITGB1) (1:100; P5D2, mouse IgG mAb, Chemicon International, Temecula, CA, USA). Alexa Fluor^TM^ 488-conjugated goat antibodies against rabbit IgG (1:500), Alexa Fluor^TM^ 488-conjugated goat antibodies against mouse IgG (1:500), Alexa Fluor^TM^ 568-conjugated goat antibodies against rabbit IgG (1:500), and Alexa Fluor^TM^ 555-conjugated goat antibodies against mouse IgM (heavy chain) (1:500) were used as the secondary antibodies and were obtained from Thermo Fisher Scientific (Waltham, MA, USA). Rhodamine-phalloidin conjugate (1:500; Thermo Fisher Scientific, Waltham, MA, USA) was used for F-actin staining, and Hoechst 33342 (1:500; Dojindo Laboratories Co., Ltd., Kumamoto, Japan) was used for nuclear staining.

### 4.4. Cholesterol Manipulation

For manipulation of cholesterol levels in cells, we depleted the cholesterol levels using MβCD and increased them using the MβCD-cholesterol inclusion complex [30]. We dissolved MβCD powder in distilled water and prepared MβCD stock solution (70 mM), which was stored at 4 °C. For the depletion of cholesterol levels, the cells were grown for 36–48 h and incubated with 1 mM MβCD dissolved in MEM containing 25 mM HEPES in 5% CO_2_/95% air at 37 °C. To increase the cholesterol levels, an MβCD-cholesterol complex (8:1 molar ratio) was prepared. First, the cholesterol powder was dissolved in prewarmed 100% ethanol (final concentration: 52 mM). Then, aliquots from the cholesterol–ethanol solution (cholesterol concentration: 10 mM) were prepared, and ethanol was evaporated at 80 °C until only white flakes of cholesterol remained. Subsequently, a solution containing 80 mM MβCD was added to the white flakes of cholesterol, and the mixture was stirred at 80 °C until cholesterol was completely dissolved in MβCD. The stock solution of the MβCD–cholesterol complex was stored at 4 °C. Next, we prepared a cholesterol-supplemented medium by adding MβCD–cholesterol complex (0.125 mM) to 25 mM HEPES containing MEM and incubated the mixture at 37 °C for 1 h in a water bath. Then, the cholesterol-supplemented medium was filtered (pore size, 0.2 µm) and added to the cells and proceeded using the same procedure as cholesterol-depleted cells. 

### 4.5. Cell Viability Assay

The cells (1 × 10^4^) were grown for 36–48 h in 96-well plates, then incubated with 100 µL of FBS-free medium (control) or cholesterol-treated medium in 5% CO_2_/95% air at 37 °C for up to 24 h. CellTiter 96^®^ AQueous One reagent (Promega Corporation, Madison, WI, USA) was added, and the absorbance was measured at 490 nm using the GloMax^®^ Discover System (Promega Corporation, Madison, WI, USA). We evaluated cell viability at different time points from 4 to 24 h.

### 4.6. Quantification of Cellular Cholesterol 

An Amplex^TM^ Red cholesterol assay kit (Thermo Fisher Scientific, Waltham, MA, USA) was used for cholesterol quantification. The cells (2 × 10^5^) were grown in a 35 mm dish for 36–48 h; then, cellular cholesterol was manipulated (as mentioned in Section 4.4) for 4 h. After washing with ice-cold phosphate-buffered saline (PBS), 200 µL of 1× reaction buffer was added to the dishes, and they were kept on ice for 25 min. The cells were scraped and centrifuged at 2300× *g* for 5 min at 4 °C. The supernatant was transferred to a tube and divided into 5 µL aliquots for the Bio-Rad protein assay (Bio-Rad Laboratories, Inc., Hercules, CA, USA), and 50 µL/well was added to a 96-well plate for cholesterol quantification. A mixture containing Amplex^TM^ Red reagent, 1× reaction buffer, horseradish peroxidase, cholesterol oxidase, and cholesterol esterase was added to each well of a 96-well plate, and the plate was incubated at 37 °C for 1 h. The fluorescence intensity was measured at 570–590 nm using MikroWin 2000 (Mikrotek Laborsysteme GmbH, Overath, Germany).

### 4.7. Fluorescence Staining

HSC-2 and HSC-3 cells were seeded on a coverslip in a 35 mm dish at a cell concentration of 0.5 × 10^5^ and grown for 36–48 h. Cellular cholesterol levels were manipulated for 4 h, as mentioned in Section 4.4, and fluorescence staining was performed. Briefly, the cells were fixed using 4% paraformaldehyde in PBS for 15 min and permeabilized using 0.2% TX in PBS at room temperature (RT) for 15 min. To block non-specific binding, the cells were incubated with 2% normal goat serum (Vector Laboratories, Inc., Burlingame, CA, USA) in PBS and reacted with primary antibodies overnight at 4 °C. The cells were then incubated with the respective secondary antibodies and Hoechst 33342 at RT for 1 h. For double staining with F-actin, rhodamine-phalloidin was also added at the time of incubation with secondary antibody. The coverslips were mounted using a mounting medium, and the cells were observed under a conventional fluorescence microscope (Eclipse E600, Nikon Corporation, Tokyo, Japan). In some experiments, a Dragonfly 505 spinning-disk confocal microscope system (Andor Technology, Oxford Instruments plc., Abingdon, UK) with a Zyla 4.2 USB 3.0 detector (Andor Technology, Oxford Instruments plc., Abingdon, UK) and a Ti2-E microscope (Nikon Corporation, Tokyo, Japan) was used to capture confocal images. For filipin III staining, the fixed cells were incubated with filipin III (100 µg/mL in PBS) at RT in the dark for 1 h, and the coverslips were mounted. For filipin III and antibody staining, the fixed cells were incubated with filipin III, then permeabilized using 0.02% TX for 10 min at RT. Afterward, the cells were incubated with primary antibody at 4 °C overnight, then reacted with secondary antibody, and the coverslips were mounted.

To evaluate CAV1 expression in a detergent-resistant membrane, TX pre-extraction of cells was performed as described in a previous study [33]. The cells were washed with ice-cold cytoskeletal-stabilizing buffer [63] and incubated on ice with 1% TX and protease inhibitor cocktail (1:50; Nacalai Tesque, Inc., Kyoto, Japan) for 30 min. Next, the cells were fixed and subjected to immunofluorescent staining without permeabilization.

### 4.8. Image Analysis

Immunofluorescence images of randomly selected cells (to include at least 50 cells) were captured from triplicate experiments. All cells, excluding those on the margins, were analyzed. Morphology was evaluated by automatic tracing of the area and perimeter of the F-actin-stained cells using ImageJ software (National Institutes of Health, Bethesda, MD, USA), and the circularity index (4π × Area/Perimeter^2^) was calculated. Confocal images were visualized using ImarisViewer 9.9.1 (Oxford Instruments plc., Abingdon, UK). The distribution of CAV1 was assessed by determining the distance between the centroid of the cell and the centroid of CAV1, as per a method reported by Zhang et al. [64]. The cells that exhibited one-sided membranous distribution of CAV1 were manually counted as cells with asymmetric membranous CAV1 using CAV1-specific immunofluorescence analysis. The cells with ITGB1 signals in lamellipodial structures or those with uneven distribution of PTEN were recognized as the cells with ITGB1 on the leading edge or PTEN on the trailing edge, respectively.

### 4.9. Timelapse Imaging and Cell Tracking Analysis

Both HSC-2 and HSC-3 cells (0.5 × 10^5^) were grown on a coverslip in a 35 mm dish. After aspiration of the old medium, cholesterol-treated medium or control medium was added, and timelapse images were taken by a CytoWatcher instrument (WSL-1800-B, ATTO Corporation, Tokyo, Japan) at 5 min intervals up to 8 h. Then, the images were tracked using NIS-Elements Advanced Research (Nikon, Tokyo, Japan), and the track plots were analyzed using the Chemotaxis and Migration tool 2.0 (ibidi GmbH, Gräfelfing, Germany).

### 4.10. Quantitative Reverse Transcription-Polymerase Chain Reaction (RT-PCR)

Total RNA was extracted from cultured cells (1 × 10^6^) using Nucleospin (Macherey-Nagel, Düren, Germany) and reverse-transcribed into cDNA using a high-capacity cDNA reverse transcription kit (Thermo Fisher Scientific, Waltham, MA, USA). A polymerase chain reaction (PCR) was performed using a specific primer for CAV1 (qHsaCID0022177, Bio-Rad Laboratories, Inc., Hercules, CA, USA) and SYBR green PCR master mix (Bio-Rad Laboratories, Inc., Hercules, CA, USA) in a QuantStudio 1 RT-PCR system (Thermo Fisher Scientific, Waltham, MA, USA). The expression of the peptidylprolyl isomerase A (PPIA, JBioS, Bio services Co.,LTD, Japan) gene was used to normalize for variance, and 5′-GCA GTA ATG GGT TAC TTC TGA AAC-3′ (forward) and 5′-TGC CTC AGG TAA TAC ATT ACA GAC-3′ (reverse) were used as primers.

### 4.11. Western Blotting

Cell lysates were prepared using an UltraRIPA kit for lipid rafts (BioDynamics Laboratory Inc., Tokyo, Japan) according to the manufacturer’s instructions. In brief, the cells (1 × 10^6^) were seeded in a 60 mm dish and treated with cholesterol for 4 h. Then, the cells were lysed using buffer A on ice for 10 min and centrifuged at 19,300× *g* for 5 min; the supernatant yielded a detergent-soluble fraction, i.e., non-raft proteins. The pellet was washed once with buffer A, incubated with buffer B at RT for 5 min, and centrifuged at 19,300× *g* for 5 min. The B buffer lysate contained the detergent-resistant fraction and lipid raft proteins. A protease inhibitor cocktail (1:50) was added to all buffers. Both detergent-soluble and detergent-resistant lysates (10 µL each) were subjected to a BCA protein assay (Takara Bio Inc., Shiga, Japan). After adjustment for protein concentration, the samples were subjected to sodium dodecyl sulfate-polyacrylamide gel electrophoresis under reducing conditions, and the proteins were transferred to polyvinylidene difluoride membranes (Bio-Rad Laboratories, Inc.). To prevent non-specific protein binding, the membrane was blocked using 0.5% ECL blocking agent (GE Healthcare UK, Little Chalfont, UK) in 50 mM Tris-buffered saline (TBS) (pH 7.4) containing 0.1% Tween-20 (TTBS) for 1 h at RT. Then, the membranes were incubated with anti-CAV1 (1:1000; D46G3 rabbit, Cell Signaling Technology, Danvers, MA, USA) diluted with TTBS at 4 °C overnight. After washing with TTBS, the membranes were incubated with horseradish peroxidase-linked anti-rabbit IgG antibody (7074S, Cell Signaling Technology, Danvers, MA, USA) diluted 1:3000 in TTBS for 1 h at RT. The target protein bands were visualized using ECL Prime Western blotting detection reagents (GE Healthcare UK, Little Chalfont, UK). Coomassie brilliant blue staining was used for protein normalization.

### 4.12. Immunohistochemistry Staining for CAV1 in Tissue Sections

Immunohistochemistry staining of the paraffin sections was performed using the ChemMate EnVision system (Dako, Glostrup, Denmark). The sections were deparaffinized in xylene and rehydrated in ethanol. Antigens were retrieved by boiling the sections in 0.5 M EDTA (pH 8) using a pressure cooker for 10 min and under high pressure for 3 min. After cooling to RT, endogenous peroxidase was blocked by incubating the sections with 0.3% hydrogen peroxide for 10 min. The sections were incubated with the primary antibody anti-CAV1 (1:400; D46G3 rabbit, Cell Signaling Technology, Danvers, MA, USA) at 4 °C overnight. After washing with TBS, the sections were incubated with EnVision Plus (Dako, Glostrup, Denmark) at RT for 30 min. The signals were developed using 3, 3′-diaminobenzidine (Dako, Glostrup, Denmark), and the sections were counterstained using hematoxylin.

### 4.13. Immunohistochemistry Analysis

The whole microscope slides were scanned using an Olympus Virtual Transmitted Light Slide Microscope VS120-S5 (Evident, Tokyo, Japan). The images were loaded in QuPath v.0.3.2 software [65] as bright-field images. Since staining of CAV1 was observed in vascular endothelial cells in all sections, the endothelial cells were selected as positive controls. Using a thresholder command in QuPath, we measured the region of tumor cells with membranous CAV1 signals of equal or stronger intensity than those of vascular endothelial cells and calculated the ratio of tumor area with membranous CAV1 and total tumor area on each slide. The tumor cells with CAV1 membrane positivity ≥15% were classified into the high-expression group, and the tumor cells with CAV1 membrane positivity of <15% were classified into the low-expression group.

### 4.14. Statistical Analysis

Statistical analyses were performed using GraphPad Prism 6 software (GraphPad Software, Inc., La Jolla, CA, USA). Experimental data were analyzed using one-way ANOVA with post hoc Tukey test or Dunn’s test. The association between membranous CAV1 expression and patient characteristics was studied using Fisher’s exact test or the chi-squared test (*n* = 63). Survival curves were analyzed using the Kaplan–Meier method, and statistical analysis was performed using the log-rank test. Univariate and multivariate Cox regression analyses were performed using JMP 9 software (SAS, Cary, NC, USA). Statistical significance was set at *p* < 0.05.

## Figures and Tables

**Table 1:** Relationship of clinicopathological factors of the OSCC cases with membranous CAV1 expression.

Characteristic	N	Membranous CAV1 Expression		*p*-Value
		High (≥15%)	Low (<15%)	
Age				
≤Median (72)	32	9	23	
>Median (72)	31	20	11	<0.01
Gender				
Male	35	14	21	
Female	28	15	13	0.31
T-factor				
T1/T2	54	22	32	
T3/T4	9	7	2	0.07
Primary site				
Tongue	58	28	30	
Buccal mucosa	4	1	3	0.43
Gingiva	1	0	1	
Lymph node metastasis				
Negative	57	23	34	
Positive	6	6	0	<0.01
Clinical stage				
I/II	51	19	32	
III/IV	12	10	2	<0.01
Mode of invasion				
1/2/3	41	15	26	
4C/4D	22	14	8	0.06
Local recurrence				
Negative	61	27	34	0.21
Positive	2	2	0	
Regional recurrence				
Negative	50	18	32	
Positive	13	11	2	<0.01

OSCC, oral squamous cell carcinoma; CAV1, caveolin-1.

**Table 2:** Univariate and multivariate analyses of clinicopathological parameters and membranous CAV1 expression in relation to relapse-free survival in OSCC.

Variable	Category	N	Univariate Analysis		Multivariate Analysis	
			RR	*p*-Value	RR	*p*-Value
Age	≤72 >72	33 30	0.59 1.68	0.32		
Gender	Male Female	36 27	0.65 1.53	0.41		
T-factor	T1/T2 T3/T4	54 9	0.38 2.64	0.13		
Lymph node metastasis	Negative Positive	57 6	0.35 2.86	0.15		
Stage	I/II III/IV	51 12	0.4 2.53	0.11		
Mode of invasion	1/2/3 4C/4D	41 22	0.15 6.58	<0.001	0.22 4.51	<0.01
Membranous CAV1	High Low	29 34	9.16 0.1	<0.001	6.47 0.15	<0.01

CAV1, caveolin-1; OSCC, oral squamous cell carcinoma; RR, risk ratio.

## Data Availability

The data are available from the corresponding author upon reasonable request.

## Supplementary Materials

The following supporting information can be downloaded at: https://www.mdpi.com/article/10.3390/ijms24076035/s1.
