# Supplementary material for: Cholesterol Is a Regulator of CAV1 Localization and Cell Migration in Oral Squamous Cell Carcinoma
Source: Int J Mol Sci. 2023 Mar 23;24(7):6035. doi: 10.3390/ijms24076035 (PMC10093846; doi:10.3390/ijms24076035)
Supplement: Supplementary file 1 [file ijms-24-06035-s001.zip › ijms-2289811-supplementary.pdf]

Supplementary Figure 1

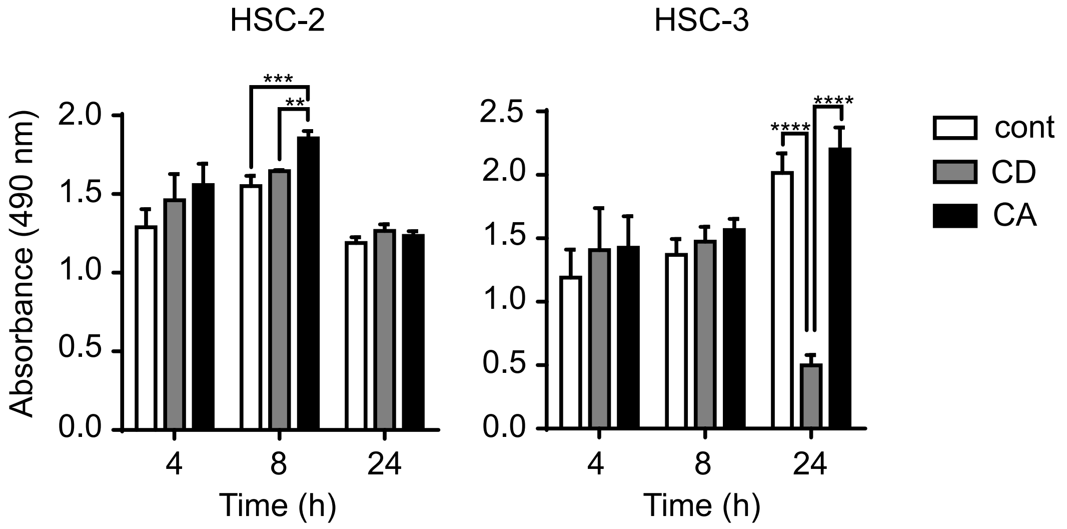

Figure S1. MTS cell viability assay of OSCC cell lines after cholesterol manipulations for 4 h, 8 h, 24 h. Open box indicates control; gray-shaded box CD; solid box CA. Triplicate results represent the absorbance of the means ± SD. $^{**}P < 0.01$, $^{***}P < 0.001$, $^{****}P < 0.0001$.

Supplementary Figure 2

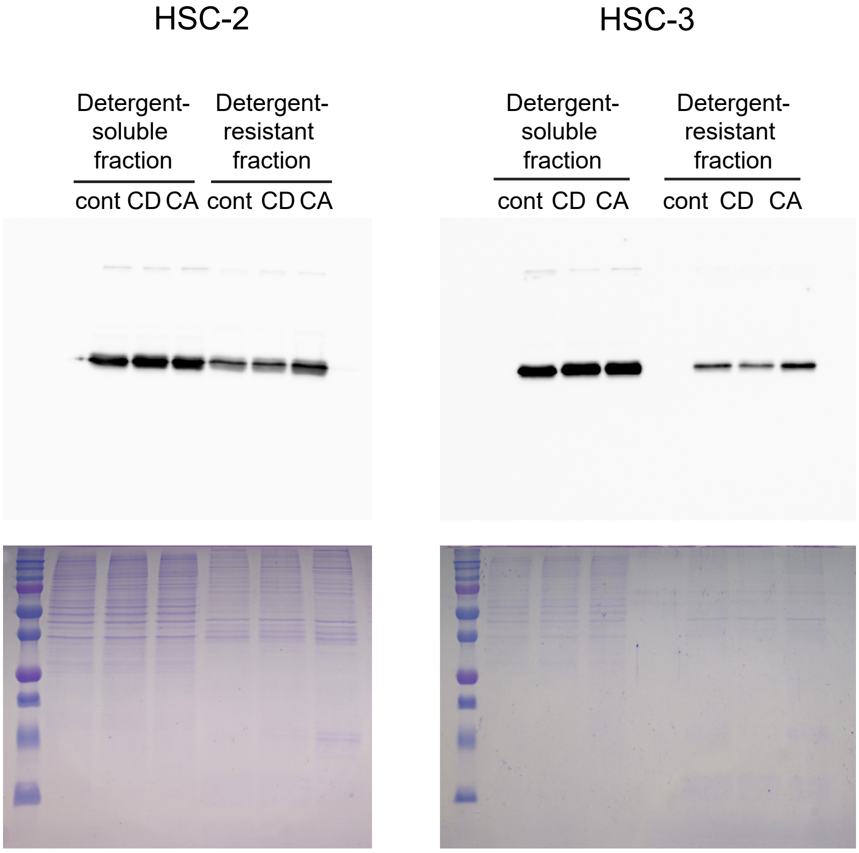

Figure S2. Western blot analysis of anti-CAV1 antibody in detergent-soluble fraction and detergent-resistant fraction. The lower panel presents Coomassie blue stain.
